# Power analysis

**DOI:** 10.7554/eLife.52232

**Published:** 2019-10-01

**Authors:** Indira M Raman

**Affiliations:** Department of NeurobiologyNorthwestern UniversityEvanstonUnited States; eLifeUnited Kingdom

**Keywords:** living science, careers in science, women in science, diversity and inclusion, research culture, power structures

## Abstract

After acknowledging that power differentials exist, can scientists find inspiration to persevere anyway?

Most of us have heard of the different experiences of men and women in science, regarding job searches, salaries, and/or peer review (Holman et al., 2018; Murray et al., 2019). For some people, supporting claims of disparity with specific measurements is itself empowering. For others, however, the simple awareness of difference, regardless of how it arose, can make us feel helpless and disempowered; it becomes a daily challenge to cope with the thought that success and power are distributed unequally in the fields that we have come to view as our homes. What I wonder, therefore, is whether we – women and men – can gain access to whatever power we actually have, right now, in a manner that might help all of us navigate the imperfect world in which we do science. It is that idea, orthogonal to the question of the existence of disparity, that I would like explore, in a wholly personal and idiosyncratic way*.

In any discussion of power in science, it makes sense first to ask, who has the power anyway? One thing I have observed: men, women, old, young, locals, immigrants, professors, trainees, people of all nations, ethnicities, creeds, and orientations – almost everyone – feels like it's not them. They conclude that power rests elsewhere – perhaps with full professors, or with men, or with native English speakers – leading to a self-perception as the 'other'. And even setting aside assumptions about which people hold the power, most scientists agree that it is not a level playing field even within a demographic group. But what is 'a level playing field'? Sometimes that phrase refers to equality of access, and working toward creating opportunity for all is, I think, obligatory. Once everyone has been admitted to the arena of possibility, however, it seems inevitable that individual variability will persist. If so, power differentials may be inescapable.

This idea can be initially disturbing, since we live in an era in which the word 'power' so often connotes exploitation. And yet, the contrary is implicit in the word 'empowerment', whose appeal is evident in our saying that we want power, presumably for constructive purposes. Are we hinting that we ourselves can handle being in charge while others cannot? Maybe old adages like 'power corrupts' tell us that managing power is troublesome for anyone. And yet, completely eliminating differentials, even in quest of an egalitarian ideal, can be just as problematic as concentrating power: at the extremes, both approaches distort to the point of promoting monoculture. And monoculture has never worked, at least not in the long term. It hasn't worked in agriculture, where repeated cultivation of the same crop has rendered the soil infertile. It hasn't worked in nutrition, where unvaried diets have led to dietary deficiencies. It hasn't worked in politics, where single-party systems have enabled oppression. It hasn't worked in economics, where depending on only one product has culminated in financial collapse. And it hasn't worked in social policy, where efforts to create a homogeneous populace have resulted in extremes of cruelty.

The opposite of monoculture is diversity in the broadest sense of this overused word. And, to my experience, a central pleasure of doing science is the diversity of the shifting roles one plays while moving through the uncharted environment of discovery. To do creative science, one holds power repeatedly but transiently, as in the child's game of 'hot potato', alternating regularly between being the teacher and being the student, the mentor and the mentored, the master and the apprentice, the reviewer and the reviewed. Power certainly resides in such pairings, but healthy relationships of this sort, which I shorthand as 'teaching relationships', can become a *benevolent* hierarchy that helps people grow rather than remain static, and cultivate learning rather than reside in ignorance. Being admitted to the tutelage of someone more knowledgeable than we are, who cares about communicating with us, is itself a treat – a privilege – that lets us reach across ranks and fields and gives us glimpses into the experiences that might lie ahead. Conversely, assuming the responsibility to be the trainer provides the reward of using our expertise for others' benefit and thereby building collegiality, understanding, and friendliness. The act of teaching transduces the scientific into the humane. It doesn't matter whether one is a formal teacher in front of a classroom or not; informal teaching situations come up constantly in the doing of science. They are not only the substrate of learning but also the fabric of community: an antidote to each person's sense of otherness.

**Figure 1:**
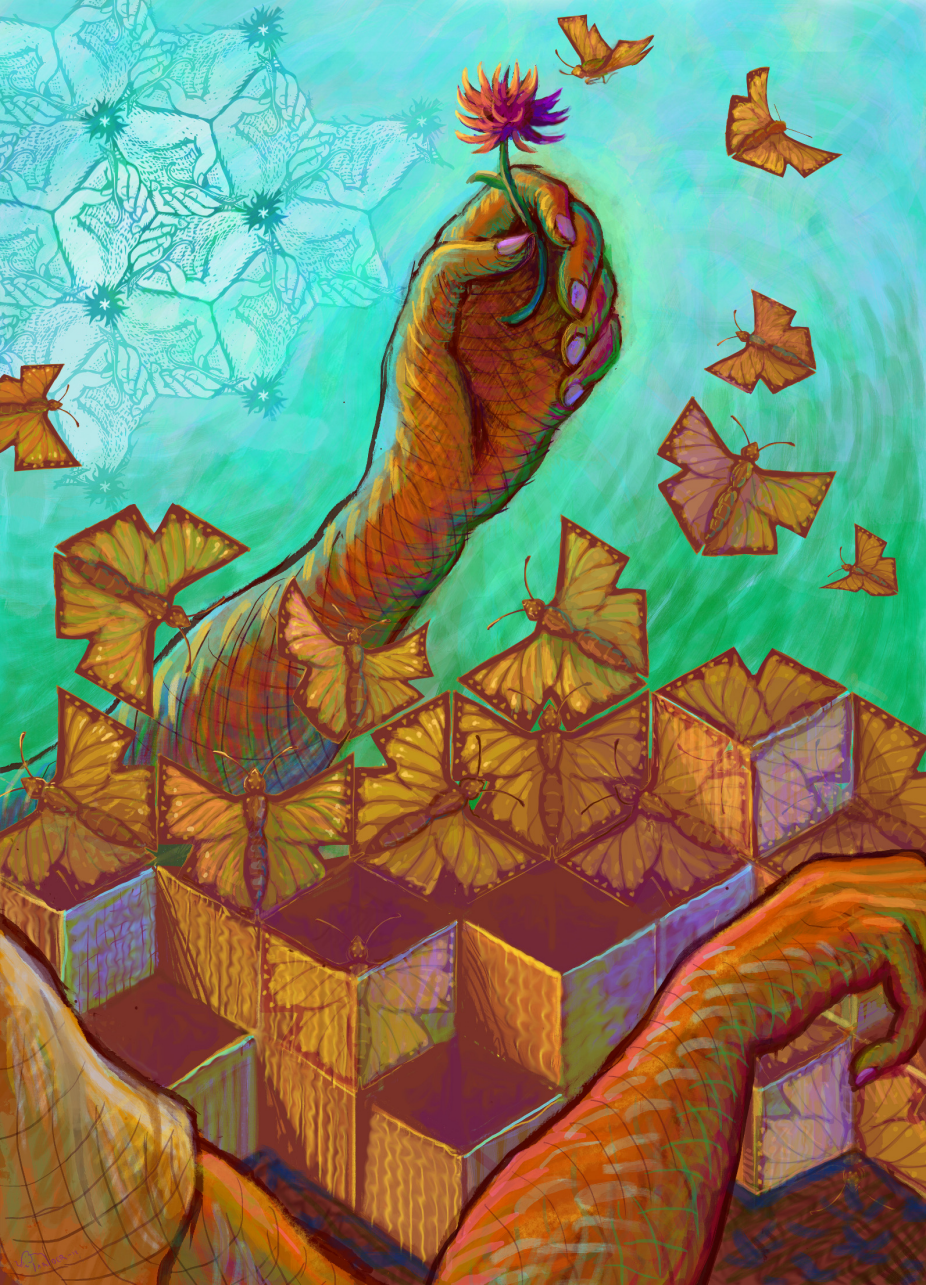
A central pleasure of doing science is the diversity of the shifting roles one plays while moving through the uncharted environment of discovery.

Illustration: Ben Marder.

Now, almost anyone who has clambered through a thesis defense, a job search, a promotion, or even just the selection process to become a reviewer or editor – and thus attained the next position of purported power – may already have found out exactly how little explicit power each next coveted position confers, regardless of whether or not one belongs to a dominant demographic group. It is rarely the kind of power in which you can tell people what to do and have them obey to the letter. If your experience parallels my own, your years as a trainee will rise up in memory and laugh at you, as you find that the people whom you thought had power, whom you had mentally characterized as walking on water, were actually treading water to keep afloat.

But you do get one thing by advancing through the ranks. You get other people's *perception* that you have power. And, wielded correctly, sometimes that is good enough. Nearly all of us can remember a teacher-figure who discouraged us; who, in a few comments or pointed words, brought us low or rattled our faith in ourselves. But almost everyone can also remember a teacher-figure who encouraged us, guided us, or gave us an awareness of our own possibility and potential. Many of us may recall some specific words that person said – words that might have been spoken in a formal moment of mentoring, or that might have been uttered as an incidental comment whose value lay in its unexpectedness. Because we believed those teacher-figures, the bad ones and the good ones, we either tumbled into dejection or found the courage and enthusiasm to press onward. And what I think is worth remembering is that, with each student you train, each colleague you advise, each review you write, *you are the teacher.* Your words and actions – sometimes the subject matter you convey and almost always the manner in which you communicate – have the potential to stay in people's memories for decades, even for the rest of their lives. So, whenever you 'teach', either face-to-face, or anonymously through a review process – each time you address a group and survey the curious or fearful or weary or cynical or apprehensive or excited or bored faces in front of you – you can be sure that every person on the receiving end is, in some way or another, hoping that something good will happen to him or her. And it is within the teacher's ability to help make that good thing happen. To me, that is power.

I actually believe that. And I'm convinced that if we can figure out the kind of power that fosters a benevolent hierarchy, we can engage in willing scientific risk in the context of cross-rank community – and open the gates to discovery. It's a beautiful idea, anyway, one that I try to practice each day. Does it always work? No. In fact, I find that a big part of being that ideal of a teacher-scientist is simply absorbing insult. Even as the teacher-figure, sometimes you do get caught on the weak end of a negative hierarchical relationship. In these cases, I have invariably found that on the other side of a seemingly calamitous process of extrication lies a life that is often good and always more compassionate. And even when no malice is at play, people who see your successes often cannot detect that you, too, are subject to threat, and they treat you carelessly. Here, you must figure out how to get past the unintended hurt, keep laughing, and find a way to love people anyway. (The polite kind of love.)

Of course, it is easy to be generous when you are secure; it is harder to remain generous when you are insecure and vulnerable – and as a scientist, one is often vulnerable, especially at grant renewal, promotion, or publication time. And because of that, it is important to remember that the reason to be a good teacher or mentor or colleague or reviewer is not because it necessarily enhances your science or your career. In fact, there will almost certainly be times when that kind of generosity will cost you in some way. The reason to be generous, despite the cost, is because humanity and interpersonal civility are the essence of civilization in general, and of scientific culture in particular, based as it is on collaboration, integrity, and constructive peer review. The core of building that kind of society is selflessness. And I mean that literally: when you do good science, the self disappears. The discoveries will stand even when the discoverer is forgotten—and we will all be forgotten, or at least misrepresented and misunderstood. Truth is independent of credit. Selflessness is often confused with service, which in turn is mistakenly equated with servitude. But selflessness isn't servitude. Selflessness is perfectly compatible with the best kinds of leadership. It simply requires a focus on the task at hand – here, research and education – rather than personal glory.

The corollary to these ideas is that anybody cannot, and need not, become anything. The notion that we can or should is a cheerful fallacy that actually promotes homogeneity. Instead, as I see it, the fact is that everybody can become something, and probably something different from the next person. This is truly the soil of diversity from which collaboration grows – each person contributing his or her talents, while depending in a healthy, often affectionate, way on each other. Learning to flip graciously between the teacher-role and student-role is the key to meaningful collaboration and benevolent hierarchy.

And although I have been talking here about power dynamics between people, the same ideas pertain to the teaching within oneself. One of the most extraordinary capacities of the human brain is its ability to compartmentalize. It has given rise to terrible hypocrisies, but it has also allowed people to do beautiful, magnanimous things in the face of personal grief and turmoil. And the internal dialogue is actually the source from which scientific research springs: teaching is transitive, asking how do I help them learn something; research is reflexive, asking how do I help myself learn something. Being a good teacher internally – giving oneself appropriate correction for one's errors and accurate credit for one's achievements – develops honesty within, which cultivates rationality without. It is the only way to create an environment of trust. And, as far as I can tell, there is no better place to do science.

## Footnote

*This essay is based on a Power Hour talk delivered at the 2019 Gordon Research Conference on the Cerebellum, which was held in Les Diablerets, Switzerland. The Power Hour is designed to address challenges women face in science and issues of diversity and inclusion.
